# HIV prevention programme with young women who sell sex in Mombasa, Kenya: learnings for scale‐up

**DOI:** 10.1002/jia2.25969

**Published:** 2022-08-26

**Authors:** Parinita Bhattacharjee, Abednego Musau, Griffins Manguro, Patricia Ongwen, Jane Mutegi, Japheth Kioko, Lisa Lazarus, Shajy Isac, Helgar Musyoki, Jan Hontelez, Daniel Were

**Affiliations:** ^1^ Institute for Global Public Health University of Manitoba Winnipeg Canada; ^2^ Partners for Health and Development in Africa Nairobi Kenya; ^3^ Jhpiego Nairobi Kenya; ^4^ International Centre for Reproductive Health Mombasa Kenya; ^5^ National AIDS and STI Control Programme Ministry of Health Nairobi Kenya; ^6^ Department of Public Health Erasmus MC Rotterdam Netherlands; ^7^ Heidelberg Institute of Global Health Heidelberg University Heidelberg Germany

**Keywords:** young women who sell sex, Kenya, key populations, HIV prevention, intervention, policy

## Abstract

**Introduction:**

In 2018, the National AIDS and sexually transmitted infection (STI) Control Programme developed a national guidelines to facilitate the inclusion of young women who sell sex (YWSS) in the HIV prevention response in Kenya. Following that, a 1‐year pilot intervention, where a package of structural, behavioural and biomedical services was provided to 1376 cisgender YWSS to address their HIV‐related risk and vulnerability, was implemented.

**Methods:**

Through a mixed‐methods, pre/post study design, we assessed the effectiveness of the pilot, and elucidated implementation lessons learnt. The three data sources used included: (1) monthly routine programme monitoring data collected between October 2019 and September 2020 to assess the reach and coverage; (2) two polling booth surveys, conducted before and after implementation, to determine the effectiveness; and (3) focus group discussions and key informant interviews conducted before and after intervention to assess the feasibility of the intervention. Descriptive analysis was performed to produce proportions and comparative statistics.

**Results:**

During the intervention, 1376 YWSS were registered in the programme, 28% were below 19 years of age and 88% of the registered YWSS were active in the last month of intervention. In the survey, respondents reported increases in HIV‐related knowledge (61.7% vs. 90%, *p* <0.001), ever usage of pre‐exposure prophylaxis (8.5% vs. 32.2%, *p* < 0.001); current usage of pre‐exposure prophylaxis (5.3% vs. 21.1%, *p*<0.002); ever testing for HIV (87.2% vs. 95.6%, *p* <0.04) and any clinic visit (35.1 vs. 61.1, *p* <0.001). However, increase in harassment by family (11.7% vs. 23.3%, *p*<0.04) and discrimination at educational institutions (5.3% vs. 14.4%, *p*<0.04) was also reported. In qualitative assessment, respondents reported early signs of success, and identified missed opportunities and made recommendations for scale‐up.

**Conclusions:**

Our intervention successfully rolled out HIV prevention services for YWSS in Mombasa, Kenya, and demonstrated that programming for YWSS is feasible and can effectively be done through YWSS peer‐led combination prevention approaches. However, while reported uptake of treatment and prevention services increased, there was also an increase in reported harassment and discrimination requiring further attention. Lessons learnt from the pilot intervention can inform replication and scale‐up of such interventions in Kenya.

## 1 INTRODUCTION

There is a growing evidence that HIV risks and vulnerabilities may be higher among young women who sell sex (YWSS) and those new to sex work than in their older counterparts [1, 2, 3, 4, 5, 6]. In 2018, there were an estimated 15,000 female sex workers (FSWs) <below 18 years of age in Kenya [7]. Mapping and size estimation data from the Transitions study [8] conducted in sex work venues in Mombasa, Kenya, have shown that 52% of all estimated FSWs were 14–24 years old [9], with an HIV prevalence of 10% among self‐identified YWSS compared to 4% among non‐YWSS of the same age [10]. The same study also revealed that YWSS in these venues reported higher experience of violence compared to those who were not selling sex [11].

Despite large population size estimates, high HIV prevalence and structural vulnerabilities, YWSS have been largely invisible within programmatic initiatives to prevent HIV among FSWs [12]. In Kenya, even though the HIV prevention programme with FSWs has scaled up in the last decade [13, 14], data from FSW programmes in Mombasa showed that the enrolment of FSWs <24 years was lower in the programme and among those enrolled, a higher proportion was lost at each service delivery step compared to their older counterparts [15]. Only 13.7% of the YWSS in the Transitions study reported contact with the local HIV prevention programme that was operational in the same venues where the study was conducted [16]. While there is evidence for effective interventions to prevent and treat HIV infections among adult FSWs [17, 18, 19, 20], less is known about the delivery of these interventions with YWSS globally and in Africa [21]. There is need to use this existing evidence to prioritize YWSS in FSW interventions and adapt and evaluate effective interventions for FSWs to address the needs and priorities of YWSS [16].

The National AIDS and sexually transmitted infection (STI) Control Programme (NASCOP), under Ministry of Health in Kenya, through consultation with young key populations, stakeholders and review of evidence, developed programme guidelines to work with young key populations in 2018 [22]. Following that, NASCOP, in partnership with Jhpiego, University of Manitoba, Partners for Health and Development in Africa and International Centre of Reproductive Health—Kenya (ICRH‐K), initiated a 12‐month pilot intervention with cisgender YWSS in Mombasa, to reduce their risk and vulnerability to HIV through provision of comprehensive combination prevention services using a YWSS peer‐led approach. We assessed the effectiveness and feasibility of the pilot intervention after 1 year, using a mixed‐methods study design. The findings of the study are presented in this paper.

## 2 METHODS

### 2.1 Study site

The study was conducted in Kisauni sub‐county, one of the six sub‐counties in Mombasa, Kenya. Key population size estimation conducted in 2014 showed that Kisuani had the highest number of YWSS compared to other sub‐counties (41% of the total estimated YWSS population) [8]. ICRH‐K, the implementing partner in the project, has a long‐standing HIV prevention programme with FSWs in the county. This pilot intervention was embedded within the existing intervention and was implemented in 12 wards of Kisauni, covering 104 FSW venues (i.e. street, sex dens, lodge, bar and public place).

### 2.2 Intervention

**Figure 1:**
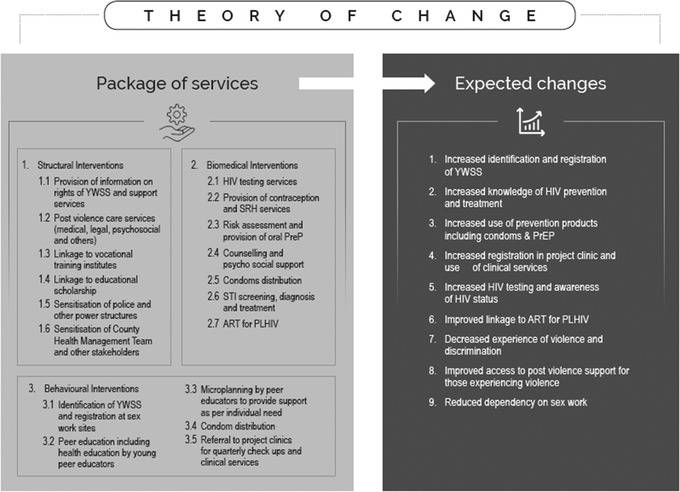
Theory of change for the intervention.

For the pilot intervention, the *theory of change* (Figure 1) proposed that provision of a combination package of structural, behavioural and biomedical services to YWSS will reduce their HIV‐related risk and vulnerability. The theory of change was guided by NASCOP's programme guidelines for work with young key populations [22]. The intervention is described in Appendix S1.

The intervention expected nine outcomes: (1) increased identification and registration of YWSS at outreach; (2) increased knowledge of HIV prevention and treatment; (3) increased use of prevention products—condoms and pre‐exposure prophylaxis (PrEP); (4) increased enrolment and use of clinical services; (5) increased HIV testing; (6) increased linkage to antiretroviral therapy (ART); (7) decreased experience of violence and discrimination; (8) improved access to post‐violence support; and (9) decreased dependency on sex work.

### 2.3 Participants

The inclusion criteria for participants in the pilot project were: all young women aged 15–24 years, who self‐identified as a cisgender FSW operating from a sex work venue. The age group of the study participants aligns with the age definition of YWSS in the national guidelines [21].

### 2.4 Data sources and analysis

#### 2.4.1 Routine programme monitoring data

Routine monthly programme monitoring data were collected from October 2019 to September 2020 by ICRH‐K to measure the outcomes 1, 4, 5 and 6. During registration at outreach, unique identification codes were assigned to YWSS. Service utilization data were collected for each YWSS using NASCOP tools [23]; the data were entered into a monthly report in Microsoft Excel. A non‐individualized aggregate report was submitted monthly by the project to NASCOP. Using the monthly aggregate report, descriptive analysis was conducted for specific indicators of interest.

#### 2.4.2 Polling booth surveys

Polling booth surveys (PBS) were conducted in the first month of the intervention in October 2019 (round 1) and after completion of the intervention in November 2020 (round 2). PBS is a group interview method in which participants are provided a private space or booth containing colour‐coded “yes,” “no” and “not applicable” ballot boxes and a set of numbered “voting” tokens corresponding to each questionnaire item. Participants then answer survey questions that the researcher reads aloud by placing the appropriately numbered token in the relevant box. This survey method is described in more detail elsewhere [24, 25]. The purpose of the survey was to measure the outcomes 2, 3, 4, 5, 7, 8 and 9. The target sample size was 91 YWSS, based on a power calculation to be able to significantly detect changes in condom use by 20% or more at 95% confidence level. Participants were divided over a total of nine PBS sessions with an average of 10 YWSS per session. The first and second round of PBS were conducted with 94 respondents and 90 respondents, respectively, out of which 38% and 39% were aged 15–19 years, respectively. The remaining respondents were aged 20–24 years. A stratified random sampling methodology was used to recruit the survey participants. Descriptive analysis was performed to produce proportions and comparative statistics. Chi‐square tests of significance were conducted to assess changes between rounds 1 and 2. The sampling method and analysis is described in detail in Appendix S2.

## 3 Qualitative data

Key informant interviews (KIIs) and focus group discussions (FGDs) with YWSS, peer educators, clinicians and programme staff were conducted pre‐ and post‐intervention (September 2019 and November 2020). The purpose of KIIs and FGDs was to understand the feasibility of implementing a comprehensive HIV prevention and treatment programme with YWSS. Six FGDs with an average of 10 participants per group, and four KIIs were conducted (three FGDs and two KIIs at pre‐ and post‐intervention). The qualitative assessment employed purposive sampling techniques. FGD participants sampled in each round included 12 unregistered, 33 registered YWSS and 17 peer educators. KII participants included two clinicians and two programme staff. Informed consent was obtained prior to the participation. FGDs and KIIs were conducted at the drop in centre (DICE) by a trained female qualitative researcher and a female note taker predominantly in Swahili using semi‐structured guides. All the FGDs and KIIs were digitally recorded, transcribed verbatim and translated into English. Field notes were typed in Microsoft Word. Two analysts reviewed the transcripts and notes and developed a codebook with clearly defined codes by mutual agreement. Thematic analysis was conducted with the aid of NVivo 11.0.

### 3.1 Ethics approval

Ethical approval for the study was obtained from the Kenyatta National Hospital/University of Nairobi Ethics and Research Committee (P268/04/2019).

## 4 RESULTS

### 4.1 Routine programme monitoring data

By the end of the 12 months intervention, the project registered 1376 YWSS (83% increase since the first month of intervention), of which 28% (383/1376) were <19 years of age (Table 1). YWSS who were engaged regularly in the intervention increased from 51% (382/750) in the first month to 88% (1209/1376) in the last month. Eighty‐seven percent (1195/1376) of YWSS registered, received clinical services and tested for HIV during the intervention. Of those who tested for HIV, 34% (401/1195) were first‐time testers, 26 YWSS were identified as living with HIV and all were linked to treatment. An increase in linkage to treatment was observed from 75% in the first month of intervention to 100% in the last month.

**Table 1:** Reach and coverage of YWSS during 12 months of the project by programme components in Mombasa, Kenya, 2020

Indicators	Month 1 (October 2019)	Month 12 (September 2020)
**1. Registration and monthly contact**	*N* = 750	*N* = 1376
1.1 Cumulative registered YWSS	750	1376
1.2 Cumulative registered YWSS below 19 years	76 (10%)	383 (28%)
1.3 Monthly contact with YWSS	382 (51%)	1209 (88%)
**2. Clinic attendance**	*N* = 750	*N* = 1376
2.1 Cumulative YWSS who attended clinic	20 (3%)	1195 (87%)
2.2 Cumulative YWSS who attended clinic below 19 years	9 (1%)	394 (27%)
**3. HIV testing**	*N* = 20	*N* = 1195
3.1 Cumulative tested for HIV	20 ( 100%)	1195 (100%)
3.2 First time testers	20 (100%)	401 (34%)
3.3 Diagnosed with HIV (including known positive)	12	26
**4. Linkage to ART**	*N* = 12	*N* = 26
4.1 PLHIV linked to ART	9 (75%)	26 (100%)

### 4.2 PBS

Comparing round 1 PBS results with round 2, the proportion of respondents exchanging sex with a paying client in the last 1 month (80.9% vs. 77.8%, *p* = 0.61), having a regular partner or lover (73.4% vs. 77.8%, *p* = 0.49), having penetrative anal sex in the last 3 months (10.6% vs. 13.3%, *p* = 0.58) and injecting narcotic drugs in the last 3 months (2.1% vs. 6.7% *p* = 0.14) remained non‐significant (Table 2).

**Table 2:** Behavioural, biomedical and structural outcomes among YWSS in two rounds of PBS in Mombasa, Kenya, 2020

	Round 1 (October 2019)	Round 2 (November 2020)	*p* Value
**Risk profile**	*N* = 94 (%)	*N* = 90 (%)	
Percent exchanging sex with a paying client in the last 1 month	76 (80.9)	70 (77.8)	0.608
Percent who have a regular partner or lover	69 (73.4)	70 (77.8)	0.492
Percent having penetrative anal sex in the last 3 months	10 (10.6)	12 (13.3)	0.575
Percent injecting narcotic drugs in the last 3 months	2 (2.1)	6 (6.7)	0.135
Percent relying solely on sex work for day‐to‐day living	34 (36.2)	49 (54.4)	0.015
**Behavioural outcomes**			
*HIV‐related knowledge*			
Percent with knowledge of having penetrative sex with a man without a condom increases the risk of contracting HIV	58 (61.7)	81 (90.0)	0.001
Percent with knowledge of using ARVs consistently by HIV‐positive individuals and being virally suppressed reduce the risk of transmitting HIV	34 (36.2)	44 (51.1)	0.044
*Condom use*			
Percent used condom at last sex with paying client	68 (72.3)	73 (81.1)	0.163
Percent used condom at last sex with regular partner or boyfriend or lover (*N* = 69 at baseline and *N* = 70 at end line)	33 (47.8)	33 (47.1)	0.936
Percent reported condom breakage or slippage at last sex	15 (16.0)	15 (16.7)	0.897
*PrEP use*			
Percent ever used PrEP for HIV prevention	8 (8.5)	29 (32.2)	0.001
Percent currently use PrEP for HIV prevention	5 (5.3)	19 (21.1)	0.002
*Contraception use*			
Percent currently use contraception for birth control	46 (48.9)	53 (58.9)	0.179
*Contact with a peer educator*			
Percent met by a peer educator from the intervention in the last 3 months	66 (70.2)	59 (65.6)	0.500
**Biomedical outcomes**			
*Clinic visit*			
Visited or receive services from the intervention/clinic/DIC in the last 3 months	33 (35.1)	55 (61.1)	0.001
*HIV testing*			
Ever taken an HIV test	82 (87.2)	86 (95.6)	0.048
Taken an HIV test in the last 3 months	52 (55.3)	61 (67.8)	0.086
**Structural outcomes**			
*Experience of violence*			
Beaten or physically forced to have sexual intercourse with someone in the last 3 months	12 (12.8)	7 (7.8)	0.269
Arrested or beaten up by police and/or city askaris when doing sex work in the last 3 months	14 (14.9)	10 (11.1)	0.448
Harassed or beaten up because of doing sex work by members of community or public like church members or other patrons in the last 3 months	13 (13.8)	16 (17.8)	0.464
Harassed or beaten up because of doing sex work by members of your family in the last 3 months	11 (11.7)	21 (23.3)	0.040
*Experience of stigma and discrimination*			
Experienced discrimination by health service providers because of sex work in the last 3 months	8 (8.5)	10 (11.1)	0.554
Experienced discrimination by education institution because of sex work in the last 3 months	5 (5.3)	13 (14.4)	0.040
*Violence and discrimination‐related support and response*			
Received support from intervention/DICE/clinic when experienced violence, harassment and discrimination in the last 3 months (*N* = 20 at baseline and *N* = 31 at end line)	8 (40.0)	18 (58.1)	0.223

Abbreviations: ARV, antiretroviral treatment; DICE, drop in centre; PReP, pre‐exposure prophylaxis.

Knowledge about HIV prevention and treatment increased significantly (61.7% vs. 90.0%; *p*<0.001 and 36.2% vs. 51.1%; *p* = 0.04, respectively). However, condom use with last paying client (72.3% vs. 81.1%; *p* = 0.16), condom use with regular partner (47.8% vs. 47.1%, *p* = 0.94) and condom breakage or slippage (16.0% vs. 16.7%, *p* = 0.89) did not change.

Both ever used PrEP (8.5% vs. 32.2%, *p* <0.001) and currently using PrEP (5.3% vs. 21.1%, *p* < 0.002) increased between rounds. Contraception use (48.9% vs. 58.9%, *p* = 0.17) and contact by peer educator in the last 3 months remained almost the same (70.2% vs. 65.6%, *p* = 0.17). A higher proportion of respondents reported visiting the project clinic in the last 3 months (35.1% vs. 61.1%, *p* < 0.001) and ever testing for HIV (87.2% vs. 95.6%, *p*< 0.04).

There were no significant changes in respondents reporting client violence in the last 3 months (12.8% vs. 7.8%, *p* = 0.26), police harassment in the last 3 months (14.9% vs. 11.1%, *p* = 0.44), being harassed or beaten by members of the community or public because of doing sex work (13.8% vs. 17.8%, *p* = 0.46) or experiencing discrimination by healthcare providers because of sex work (8.5% vs. 11.1%, *p* = 0.55). A slightly higher proportion of YWSS reported being harassed or beaten by family members because of doing sex work in the last 3 months (11.7% vs. 23.3%, *p* < 0.04) and experiencing discrimination by educational institution in the last 3 months (5.3% vs. 14.4%, *p* < 0.04). There were no significant changes in respondents reporting receiving support from the intervention to address these experiences of violence and discrimination (40.0% vs. 58.1%, *p* = 0.22). In round 2, respondents who reported relying solely on sex work for day‐to‐day living increased (36.2% vs. 54.4%, *p* < 0.01).

### 4.3 Qualitative data

#### 4.3.1 Early signs of project success

YWSS reported increased awareness and knowledge of prevention interventions, including proper use of condoms, PrEP and post‐violence support. Further, YWSS reported improved access to services, including HIV testing, treatment for SSTIs, contraceptives, condoms and PrEP.

*Before I came here, I didn't know anything about health, about condom, about PrEP*.

*But since I came, I came to know about PrEP or when someone rapes you out there, you can get help, (22‐year‐old, YWSS)*

The project worked with YWSS peer educators who conducted mobilization within their social networks at the sex work venues. YWSS reported that they identified with the YWSS peer educators and this helped establish trust and increased the project's acceptability and uptake of services. Additionally, the DICE offered a safe space to receive services with non‐judgemental providers and comprehensive services and facilitated a social connection with fellow YWSS through group sessions.

Inclusion of non‐health services also encouraged YWSS to access services. Upon receipt of vocational skills training, some respondents started their own businesses and created a saving culture, which has contributed towards a supplementary source of income.

*They [YWSS] have also been trained on microfinance and they've also started a merry‐go‐round, where they contribute 1000 shillings every month and give it to one person. We have like three groups and the peer educators have also started a chamaa (informal savings group) where they contribute twenty shillings daily and give it to one person and this person can start up some small business, (programme staff, pilot project)*

The respondents attributed the project's success to the YWSS‐centric project implementation model, which was responsive to their feedback. YWSS were engaged routinely in planning the pilot project to increase its responsiveness towards their needs.

*Young KPs [YWSS] are the ones who are supporting us to do the implementation framework, so we have them in our planning…They are also part and parcel in terms of following‐up and ensuring that implementation is happening and probably monitoring of the same, (programme staff, pilot project)*

#### 4.3.2 Missed opportunities for optimization

The pilot engaged various stakeholders. Activities were conducted at sex work venues and family influences were underestimated. On a few occasions, parents or guardians came to the DICE with complaints about the provision of condoms and contraceptives. The respondents also recommended that future programs adopt a proactive stakeholder engagement strategy with parents and relevant government authorities.

*They [families] allege that we are spoiling young girls; giving them condoms, family planning services, and PrEP. They believe that the girls have not started sex work and are very innocent. They are virgins, (programme staff, pilot project)*

Navigating legal hurdles surrounding the provision of contraception to YWSS <18 years posed another challenge. Though YWSS were identified from sex work venues and most were mature minors, there were two instances where guardians reported the provision of contraceptives by the project to the police. Such incidences made service providers uneasy about providing services to YWSS below the legal age, even if the YWSS needed them.

*“As a clinician I see so many young KPs [YWSS] coming to the DICE and they have been mobilized from the hotspots [sex work venues]… it's not that they have been mobilized from their houses or from home. So, when they ask for family planning services and am not able to offer…it becomes a challenge even explaining to them the reason why we are not offering them family planning services”, (female clinician, pilot project)*

It was not always possible to continuously reach some YWSS because they did not possess phones, were mobile and moved between sex work venues or were denied permission by sex den management to attend the DICE activities and services. Some YWSS wanted their sex work identity to remain anonymous and feared accessing services from the DICE, which was popularly identified as a sex worker clinic.

*It becomes so hard for one to come here [DICE] because they think that if they come here, when they get out and someone who sees them will judge them for the kind of work that they do. It becomes so hard, it is hard, (19‐year‐old YWSS)*

The pilot could not meet all the expectations of YWSS. Many YWSS wanted scholarship opportunities to pursue formal education but the pilot could identify very few matching opportunities.

*So, we tried to get a number of them into TVET [technical and vocational education and training] centre for vocational training but I think the qualifying criteria for TVET were too high so when we looked at the qualifications, none of the girls who showed interest actually made it through, (programme staff, pilot project)*

## 5 DISCUSSION

To the best of our knowledge, our paper is the first to report on results of delivering a comprehensive HIV prevention intervention with YWSS in Kenya. It should be noted that during the intervention period (October 2019–September 2020), Kenya also experienced the COVID‐19 pandemic. Government of Kenya implemented aggressive measures recommended by the World Health Organization to proactively limit the spread of COVID‐19 from March to June 2020 [26]. These measures included rigid and abrupt stay‐at‐home policies enforced through curfews and lockdowns [26]. HIV prevention and treatment interventions, especially peer‐led outreach, were disrupted for 4–6 months and programmes made critical adaptations to ensure services were available for FSWs.

Over the 12 months period, registration of YWSS, clinic visits and utilization of services increased despite COVID‐19‐related restrictions. The project has been also successful in ensuring that YWSS <19 years access services. Respondents reported increases in HIV‐related knowledge, ever and current usage of PrEP, HIV testing and linkage to treatment. YWSS appreciated the comprehensive, non‐judgemental and safe services offered under one roof, peer support and social connection with other YWSS, including the involvement of YWSS in planning and implementation. However, increase in dependency on sex work, harassment by family and discrimination at educational institutions was also reported. Certain behaviours related to condom use and contact with a peer educator did not improve as expected. We think that some of the unexpected results may have been a result of COVID‐19 during which peer‐led outreach to FSW venues was disrupted. YWSS may have also experienced challenges balancing using HIV prevention methods and staying resilient during the pandemic with reduced income, limited access to prevention options and reduced peer support [26, 27]. The time frame of the intervention may have been short to change some of the structural outcomes like dependency on sex work and have to be explored further to determine its priority in interventions with YWSS [28, 29, 30].

Our findings are similar to the findings reported by Busza et al. about their pilot intervention with YWSS in Zimbabwe, where they were successful in reaching higher YWSS with acceptable services [31]. Chabata et al. in their study from Zimbabwe also found that YWSS who were engaged in interventions reported an increase in knowledge about HIV prevention and treatment and initiation and continuation on PrEP by intervention end [32]. Another study from Burkino Faso [33] has also shown that peer‐led interventions and involvement of the community had a positive impact on reduction of risky behaviours among YWSS. Delany‐Moretlwe et al. in their review of health services recommended that young key populations require comprehensive, integrated services that respond to their specific developmental needs, including health and non‐health services within the context of a human rights‐based approach [34].

Similar to other studies, our project experienced specific challenges, including following‐up with mobile YWSS, fear of some YWSS being identified if they visited the DICE and the expectation to address a wide range of non‐HIV needs [31]. In addition, the legal framework in the country is not supportive of providing sexual and reproductive health services to girls <18 years and criminalizes sex work. These challenges need to be addressed to scale‐up HIV prevention interventions for YWSS. The pilot engaged various stakeholders, but comprehensive and continuous engagement of parents of YWSS was not adequately done.

Our study shows that a comprehensive YWSS peer‐led intervention using a combination prevention approach involving YWSS in design and implementation can ensure that YWSS access information, prevention and treatment services to reduce their risk and vulnerability to HIV. For future replication and scale‐up, the implementing partners need to involve key stakeholders like family members, community leaders, social support services, child rights groups and law enforcement agencies to support the interventions. The intervention should also build strong partnerships with government healthcare providers to make services accessible for YWSS who do not wish to access the DICE and partner with other programmes like DREAMS [35] in the county to address the non‐HIV needs of the population. The programmes also need to advocate for legal reforms to improve access to sexual and reproductive health services for young people and those who sell sex.

The study has several limitations: (1) the data are from a cross‐sectional survey and cannot be used to determine causality; (2) the responses to the survey were self‐reported and may have some social desirability bias though the PBS method adopted has less bias compared to face to‐face interviews [36]; (3) the respondents may have had some social desirability bias related to identifying themselves as FSWs; and (4) the monitoring data and survey data analysed captured data at an aggregated level and did not allow individualized analysis. One of the strengths of the study is the use of multiple data sources.

## 6 CONCLUSIONS

Our 1‐year pilot intervention successfully rolled out peer‐led HIV prevention services for YWSS and improved critical population‐level outcomes. The study shows that when supported by policy guidance, embedding YWSS‐led interventions within larger FSW interventions is feasible and can be scaled‐up with inclusion of a few critical learnings. Lessons learnt from implementation of this project can be used to scale‐up interventions with YWSS in Kenya.

## COMPETING INTERESTS

The authors declare that they have no competing interests.

## AUTHORS’ CONTRIBUTIONS

PB, AM and DW conceptualized the paper. PB, JK and SI designed the plan of quantitative analysis. AM and DW designed the qualitative aspect of the study and led the analysis. PB and AM wrote the first draft of the paper with edits from HM. PB, AM, HM, JM, PO and GM contributed to questionnaire design, and interpretation of data and results. JK and AM contributed to the data collection. JH, LL and DW reviewed the manuscript and provided edits and suggestions. All authors have read and approved the final manuscript.

## FUNDING

This study is made possible by the support of the Bill & Melinda Gates Foundation (BMGF) under grant INV‐007340.

## DISCLAIMER

The views expressed herein are those of the authors and do not necessarily reflect the official policy or position of BMGF.

## Supporting information




**Appendix S1**. The pilot intervention description

**Appendix S2**. Sampling and analysis

## Data Availability

This data is confidential considering the fact that YWSS are a criminalized population in Kenya and sharing names of sites and individual information may put their life in danger. Aggregate level de‐identified data tables are available and the corresponding author (bhattacharjee.parinita@gmail.com) will be able to facilitate access to the data. A formal request needs to be made and a data sharing agreement will be signed before sharing the data.
