# Supplementary material for: HIV prevention programme with young women who sell sex in Mombasa, Kenya: learnings for scale‐up
Source: J Int AIDS Soc. 2022 Aug 26;25(8):e25969. doi: 10.1002/jia2.25969 (PMC9418418; doi:10.1002/jia2.25969)
Supplement: Supplementary file 1 — Appendix S1. The pilot intervention description [file JIA2-25-e25969-s001.docx]

**Appendix 1. The pilot intervention description**

Guided by the theory of change, the pilot intervention in Mombasa engaged the YWSS in consultation to refine the intervention design as stated in the guidelines, to suit their needs and context. Based on the consultations, the intervention engaged and trained (using the national curriculum for peer educators) 10 YWSS peer educators to specifically identify, reach and register new YWSS, provide information, distribute and demonstrate condoms, provide post violence support, mobilise and refer YWSS to access clinical services. The YWSS peer educators were supported by two outreach workers who provided supportive supervision to maintain fidelity of the intervention. The pilot intervention site in Kisauni subcounty already had one drop-in-centre (DICE) which included a clinic to provide HIV services, through two clinicians. The clinicians and DICE staff were trained to understand the needs and priorities of YWSS and develop skills to provide HIV and other reproductive and sexual health services through the clinics. Specific events and group sessions were conducted at the DICE led by YWSS peer educators to provide space for YWSS peers to discuss their issues and build solidarity with other young and adult sex workers. Besides violence prevention and response support, the project also provided linkage to educational scholarships and other income generation support to reduce dependency on sex work. The project established a strong collaboration with county-level stakeholders, to create an enabling environment and establish linkages to address the non-health needs of YWSS. The stakeholder’s forum established for the pilot intervention included representation from sectors like gender and children, internal security, education (in addition to health) and met regularly to discuss progress of the intervention and address concerns related to security, linkages with social protection schemes and challenges related to policies and laws related to criminalization of sex work or age of consent.
