# Supplementary material for: HIV prevention programme with young women who sell sex in Mombasa, Kenya: learnings for scale‐up
Source: J Int AIDS Soc. 2022 Aug 26;25(8):e25969. doi: 10.1002/jia2.25969 (PMC9418418; doi:10.1002/jia2.25969)
Supplement: Supplementary file 2 — Appendix S2. Sampling and analysis [file JIA2-25-e25969-s002.docx]

**Appendix 2. Sampling and analysis**

A list of YWSS in the subcounty of intervention, was developed by the YWSS peer educators and stratified by registration status to ensure representation in the survey (66% to the registered list and 33% to the non-registered list). YWSS were registered into the programme using a national registration form [23] following reaching them at outreach and obtaining their consent to receive services from the programme. This registration process was led by the peer educators when they identified and met a new YWSS at the venues of sex work. The form was filled after the peer educators built rapport with the new YWSS and their consent was taken. This form was then submitted to the project monitoring team who entered the data in a password protected computer and generated a unique identification code (UIC) for the YWSS. A master list of registered YWSS was generated to be used as a sampling frame. A total of 60 YWSS were randomly selected from the registered list and 30 YWSS were selected from the non-registered list. The names of selected participants were provided to the YWSS peer educators to mobilise and invite them to participate in the survey. Individuals who accepted the invitation were provided with details of their PBS session, which took place in the DICE managed by ICRH-K. All consenting processes were followed as per the protocol.

Aggregate responses per PBS session were entered into Microsoft Excel and exported to SPSS 20.0 for analysis. Descriptive analysis was performed to produce proportions and comparative statistics. Chi square tests of significance were conducted to assess changes between round 1 and round 2. Data were weighted considering estimated YWSS and the sample size in each group to generate overall estimates of different indicators. Overall selection probability was derived using the total sample size and the estimated size of YWSS across the consistent locations, whereas the selection probability of registered and non-registered YWSS (stratum) was derived using the sample size and the estimated size of the key population (KP) at the respective levels. The final weights were calculated using both overall sampling probability and the sampling fraction at the stratums.
